# Structure-Based In Silico Investigation of Agonists for Proteins Involved in Breast Cancer

**DOI:** 10.1155/2022/7278731

**Published:** 2022-01-06

**Authors:** Arpita Roy, Ashutosh Anand, Saksham Garg, Mohd Shahnawaz Khan, Sidharth Bhasin, Muhammad Nadeem Asghar, Talha Bin Emran

**Affiliations:** ^1^Department of Biotechnology, School of Engineering & Technology, Sharda University, Greater Noida, India; ^2^Delhi Technological University, Rohini, New Delhi, India; ^3^Department of Biochemistry, College of Sciences, King Saud University, Riyadh, Saudi Arabia; ^4^Indian Institute of Technology Delhi, Delhi, India; ^5^Department of Medical Biology, University of Québec at Trois-Rivieres, Trois-Rivieres, Québec G9A 5H7, Canada; ^6^Department of Pharmacy, BGC Trust University Bangladesh, Chittagong 4381, Bangladesh

## Abstract

Cancer is recognized as one of the main causes of mortality worldwide by the World Health Organization. The high cost of currently available cancer therapy and certain limitations of current treatment make it necessary to search for novel, cost-effective, and efficient methods of cancer treatment. Therefore, in the current investigation, sixty-two compounds from five medicinal plants (*Tinospora cordifolia, Ocimum tenuiflorum*, *Podophyllum hexandrum*, *Andrographis paniculata*, and *Beta vulgaris*) and two proteins that are associated with breast cancer, i.e., HER4/ErbB4 kinase and ER*α* were selected. Selected compounds were screened using Lipinski's rule, which resulted in eighteen molecules being ruled out. The remaining forty-four compounds were then taken forward for docking studies followed by molecular dynamics studies of the best screened complexes. Results showed that isocolumbin, isopropylideneandrographolide, and 14-acetylandrographolide were potential lead compounds against the selected breast cancer receptors. Furthermore, *in vitro* studies are required to confirm the efficacy of the lead compounds.

## 1. Introduction

Breast cancer is a heterogeneous group of diseases that originate in the breast tissue and result in the formation of a lump or a mass in the breast. Breast cancer mostly originates from the epithelial cells lining the milk duct [1, 2]. When the tumor is small and easily treatable, no concrete symptoms are observed, therefore making early screening important. However, a major symptom is the presence of a painless lump/mass in the breast. In some cases, cancer spreads to the lymph nodes present in the underarms and can cause swelling or lumps, even though the tumor itself is not large enough to be felt by the patient. Pain in the breast, a feeling of heaviness, swelling, or redness of the skin, and spontaneous discharge from the nipples are some of the rare symptoms experienced by some patients [3]. It is the most frequent cause of cancer and cancer-related death among women worldwide. It impacts more than 2.1 million women each year. According to the World Health Organization, in 2018, more than 620 thousand women died from breast cancer worldwide. This constitutes about 15% of all cancer-related deaths among women [4]. A total of 6.9% of cancer deaths are attributed to breast cancer with 684,996 deaths in 2020.

Estrogen receptor (ER) *α*, an ER subtype, plays a major role in the physiological development of the body [5]. The reproductive, central nervous system, skeletal, and cardiovascular systems are some of the organ systems where ER*α* plays an important role in the development and functioning [5]. As shown in [5–7], ER*α* is widely expressed throughout the body such as in the uterus, mammary glands, male reproductive system, ovaries, spleen, kidney, and lungs among other organs. ER*α* is responsible for human breast cancer progression [6, 8]. Approximately 75% of breast tumors have ER*α* at the time of diagnosis (ER-positive breast cancer). Therefore, it has been selected as a marker for prognosis during the course of therapy that a patient receives. ER-positive markers have been found to have a better prognosis than ER-negative tumors. Tamoxifen is the most widely used drug for treating cases of ER-positive breast cancer when the patient requires endocrine therapy [9]. Other selective ER modulatory drugs are toremifene and raloxifene. Aminoglutethimide and exemestane are potent aromatase inhibitors for ER [10].

HER4/ErbB4 is a member protein of the epidermal growth factor receptor (EGFR) family. Each member of the EGFR family is essential for normal development [11] in animals and humans. They are necessary for the healthy development of the heart, nervous system, and mammary gland [11]. HER4 is known for its crucial role in carcinogenesis [12]. However, unlike other members of EGFR, HER4 signaling is less understood. It is known to play a positive role in cancer progression, especially breast cancer in humans [13, 14]. As reported by Zhu et al. [15], HER4, alone, could moderate the estrogen-induced growth of breast cancer cells. And, as observed *in vitro,* overexpression of HER4 influences cell cycle arrest and apoptosis significantly in breast cancer [15, 16]. Canertinib, developed by Pfizer, inhibits HER2 and HER4, but due to its limited effect, as shown in phases I and II, this drug was discontinued [17]. Afatinib, an irreversible tyrosine kinase inhibitor, simultaneously targets HER2 and HER4 [18]. It also suppresses HER3-mediated signaling [19].

Natural compounds are one of the potential sources of bioactive compounds [20]. Plant-derived compounds possess a wide range of therapeutic actions, which include anticancer, antidiabetic, antibacterial, and antifungal activities [21–23]. The anticancer activity of various plant-derived compounds has been reported in various studies [24–28]. This study elucidates the molecular docking and dynamic results between biochemical compounds taken from five different plants, namely, *Tinospora cordifolia, Ocimum tenuiflorum, Podophyllum hexandrum, Andrographis paniculata,* and *Beta vulgaris*, and two proteins, namely, human ER*α* (PDP Id: 2IOG) and HER4/ErbB4 (PDP Id: 3BBT), to propose a natural phytochemical compound which could potentially treat breast cancer.

## 2. Materials and Methods

### 2.1. Protein and Ligand

The 3D crystal structures of the proteins considered in this study were imported from the Protein Data Bank (RCSB PDB) Web server [28]. For the following study, two proteins were taken for the search of their antagonists, HER4/ErbB4 kinase (PDB ID: 3BBT) and human estrogen receptor *ɑ* (PBD ID: 2IOG).

### 2.2. Ligands

For the analysis and prediction of the potential ligand, a data set of 62 phytocompounds from *Tinospora cordifolia*, *Podophyllum hexandrum*, *Andrographis paniculata*, *Beet vulgaris*, and *Ocimum sanctum* was selected (Table 1). The structures obtained from the PubChem database [27, 29] of all compounds were converted from .sdf format to .pdb format using the Discovery Studio Visualizer by BIOVIA.

### 2.3. ADME Testing

The SwissADME tool [30] by ExPASy tools was used to determine the values of parameters proposed in the Lipinski Rule of Five [31]. In addition to lipophilicity, the number of hydrogen bond donors and accepters, and molecular weight, one parameter, molar refractivity (Ghose rule), was also taken into account to test the drug-likeliness of all the 62 compounds [32].

### 2.4. Molecular Docking

Interaction studies of receptor structure with selected ligands were performed using the AutoDock v4.2.6 interface. The protocol started with the preparation of a receptor/protein in which surrounding water was removed from the vicinity of the protein, Kollman charges were added, and polar hydrogens were added, merging nonpolar at the same time. All the rotatable bonds of the ligand were allowed to rotate, and the Gasteiger charges were computed. Grid coordinates were selected using the existing inhibitor in the receptor after which the bound inhibitor was also removed to vacate the active site. The Lamarckian GA output was used to obtain the docking results using a genetic algorithm. Of the 10 conformations obtained, the one with the least binding energy was selected, and 2D binding interactions between active site residues and the ligand were generated using the BIOVIA Discovery Studio Visualizer v19.1.0.18287.

### 2.5. Molecular Dynamics

100 ns simulations of the docked receptor-ligand complex were carried out. Molecular simulations were carried out on the Desmond–Maestro module 2020. All the parameters and algorithms were kept as default. Desmond by itself uses the most efficient algorithms for generating precise output data. The TIP3P model system of water was provided to the docked complex to provide a medium. 0.15 M Na^+^ ions were added to neutralize the whole system with OPLS-AA 2005 as the assigned force field. The SHAKE/RATTLE algorithm restricted the covalent bond movement. NVT ensemble with 300K as temperature and 1 bar as pressure, and all inputs were combined using the RESPA integrator. 100 ns simulations were allowed to be subdivided into 1000 frames for dynamic analysis of protein-ligand interaction [33, 34].

### 2.6. PASS Webserver

Based on the structures using multilevel neighbors of atoms description, the PASS webserver can be used to predict the biological activity of the compound. The SMILES of the compound are taken as the input, and the probability of a biological activity can be obtained as the output.

## 3. Result and Discussion

### 3.1. ADME Testing

A preliminary structure-based analysis was conducted on the selected 62 phytocompounds which were reported in our earlier study [35]. Compounds were subjected to a total of 5 parameters: logP/lipophilicity (<5), molar refractivity (40–130), molecular weight (<500 Da), hydrogen bond acceptor (<10), and hydrogen bond donors (<5) [36, 37]. 18 small molecules showed two or more violations of parameters which resulted in their omission from further analysis.

### 3.2. Molecular Docking

**Figure 1:**
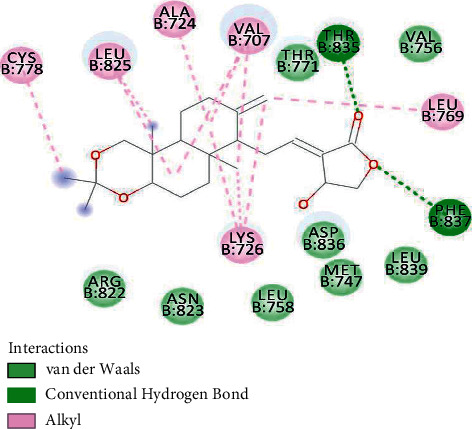
3BBT residues interacting with isopropylideneandrographolide.

The minimum binding energy among all 3BBT-isopropylideneandrographolide complexes was −9.41 kcal/mol. A total of 16 residues interacted with the ligand molecule. THR835 and PHE837 formed strong hydrogen bonds. Another type of interaction was alkyl and van der Waals. CYS778, LEU825, ALA724, VAL707, LEU769, and LYS726 formed an alkyl bond with isopropylideneandrographolide (Figure 1) (Table 2).

**Figure 2:**
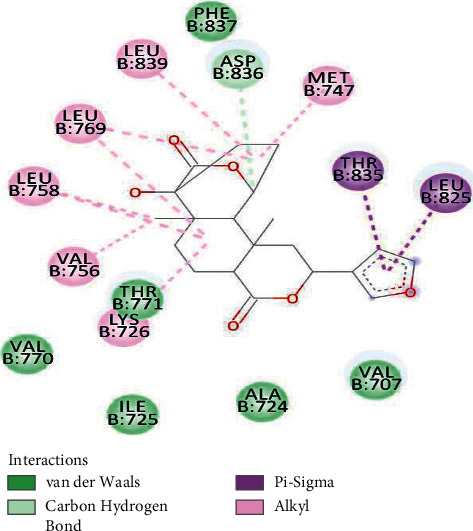
3BBT residues interacting with isocolumbin.

Out of the 10 conformations obtained for the 3BBT-isocolumbin complex, −9.06 kcal/mol was the best binding energy. Four different types of bond formation took place. THR835 and LEU 825 interacted with the five membered ring with pi-sigma interaction. ASP836 formed a conventional carbon-hydrogen bond. LYS726, VAL756, LEU758, LEU769, LEU839, and MET747 interacted by forming alkyl bonds with the ligand. The other six residues complemented these interactions with van der Waal forces (Figure 2).

**Figure 3:**
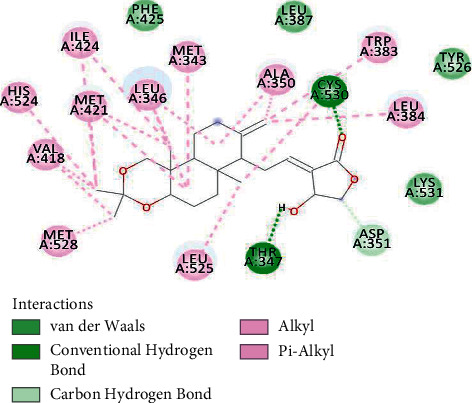
2IOG residues interacting with isopropylideneandrographolide.

Figure 3 represents the best docked conformation of the 2IOG-isopropylideneandrographolide complex having a binding energy of −10.3 kcal/mol. 18 residues in total interacted with the present ligand. CYS530 and THR347 formed conventional hydrogen bonds. ASP351 was the only residue forming a carbon-hydrogen bond. 4 residues interacted with weak van der Waals forces. All the other residues either interacted with either the alkyl or pi-alkyl bond formation.

**Figure 4:**
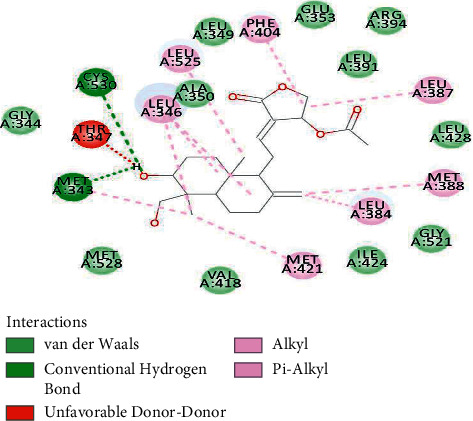
2IOG residues interacting with 14-acetylandrographolide.

The best complex with 14-actetylandrographolide showed a binding energy of −9.26 kcal/mol; cumulatively, 21 residues around the ligand interacted with it. CYS530 and MET343 acted as anchors forming strong conventional hydrogen bonds. MET421, LEU384, MET388, LEU387, PHE404, LEU525, and LEY346 formed either alkyl or pi-alkyl bonds. 11 residues showed weak van der Waals interactions. THR347 formed an unfavorable donor-donor bond (Figure 4) (Table 1).

### 3.3. Molecular Dynamics

Simulation studies were carried out on the two best docked complexes for each receptor taken into account. Each dock was allowed to simulate in the dynamic environment for 100 ns seconds to yield the potential energy and total energy (Table 3).

### 3.4. Structural Deviation and Compactness

Using four parameters, the conformational stability of the protein and ligand can be analyzed. The root mean square deviation (RMSD), root mean square fluctuations (RMSF), radius of gyration (rGyr), and solvent accessible surface area (SASA) plots can be insightful for defining the compactness of protein and ligand complexes.

#### 3.4.1. HER4/ErbB4 Kinase (3BBT)

**Figure 5:**
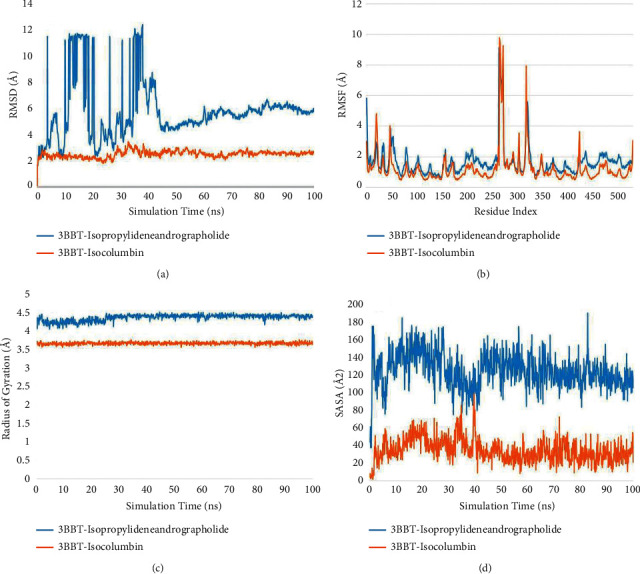
Structural and compactness analysis. (a) Root mean square deviation (RMSD) plot. (b) Root mean square fluctuation (RMSF) plot. (c) Radius of gyration (rGyr) plot. (d) Solvent accessible surface area (SASA) plot of isopropylideneandrographolide (blue) and isocolumbin (orange) with 3BBT.

A stabilized RMSD plot can be observed with isocolumbin as the ligand molecule. The graph can be observed to fluctuate a little but around a fixed average value only, i.e., 2.5 Å. Although during 20–40 ns a few small peaks are visible of magnitude 1 Å for backbone atoms and C*ɑ* atoms, this sudden peak can be regarded to the internal vibrations of the atoms. After 40 ns, a stable plateau is attained, lasting throughout the simulation. With isopropylideneandrographolide as a ligand molecule, a significant deviation (>3) can be seen (>3 Å) suggesting large conformational changes in the protein, which is not preferable. After 40 ns the graph stabilizes to the end around a value of 5 which is still higher compared to the RMSD of the 3BBT-isocolumbin complex (Figure 5(a)).

RMSF graphs for both the ligand complexes can be observed as they should be. Secondary structures like the alpha-helix and beta-sheets are more rigid portions of the protein and therefore should fluctuate less, which is exactly what the RMSF plot showed. The unstructured part, including loops and straight chains of amino acids, fluctuated relatively more (Figure 5(b)).

The rGyr plot for the isolcolumbin complex yielded a value of 3.66 ± 0.12 Å. The probability distribution graph revealed that in most frames, the 3.66 Å was achieved with little or no fluctuation overall. The isopropylideneandrographolide complex showed a higher rGyr value of 4.40 Å with a very nominal crest and troughs. Both the graphs suggest the compactness of the complex; however, the lower value of isocolumbin gives it an edge suggesting a more compact structure during the simulation course (Figure 5(c)).

Isocolumbin again provides a lower SASA value than the isopropylideneandrographolide; an average of 30 Å SASA is observed with the isocolumbin ligand, whereas this number goes up to 100 Å with the other ligand, with more variations in the overall graph. The binding of isolcolumbin with the protein is fairly more stable and the core residues are not exposed to the surrounding water, suggesting a good binding of the ligand with the 3BBT receptor (Figure 5(d)).

#### 3.4.2. Human Estrogen Receptor *ɑ* (2IOG)

**Figure 6:**
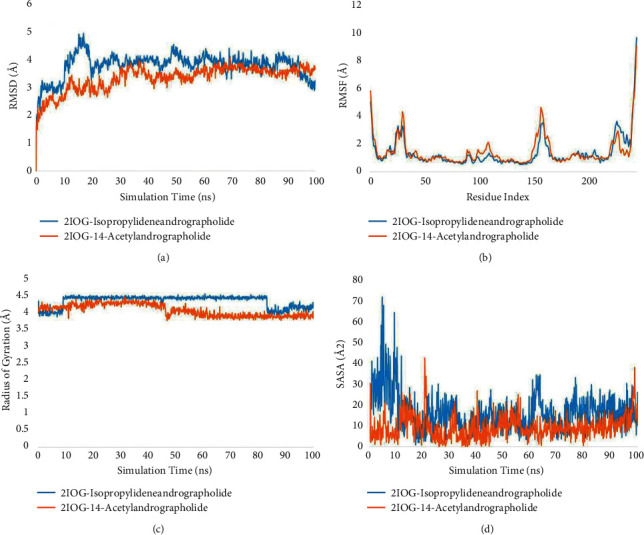
Structural and compactness analysis. (a) Root mean square deviation (RMSD) plot. (b) Root mean square fluctuation (RMSF) plot. (c) Radius of gyration (rGyr) plot. (d) Solvent accessible surface area (SASA) plot of isopropylideneandrographolide (blue) and 14-acetylandrographolide (orange) with 2IOG.

Toward the later stages of the dynamics, the RMSD plot with 14-acetylandrographolide stabilized almost at 3.2 without any considerable fluctuations. With the second ligand molecule, i.e., isopropylideneandrographolide, the graph can be observed averaging at about 3.5 Å. At the beginning, from 10 to 20 ns, a relatively higher peak can be observed, but after 20 ns, the plateau is attained. Toward the end, there was a dip in the graph as well. The observations are suggestive of protein stability with both the ligand molecules (Figure 6(a)).

Fluctuations considering residues as the basic entity were measured using the RMSF plot. The general convention of amino acids forming secondary structures deviating less as compared to free amino acids such as loops and straight chains was quite visibly followed by both ligand complexes. The binding pocket can be said to lie somewhere in the middle of being flexible and rigid as residues of both natures interacted with the ligand (Figure 6(b)).

The rGyr plot is suggestive of the compactness of the protein structure. At 100 ns, a value of 3.8 Å was obtained with the protein-14-acetylandrographolide complex, while with isopropylideneandrographolide a value of 4.2 Å was obtained. The smaller value of 2IOG-14-acetylandrographolide suggests a more compact protein (Figure 6(c)).

Both the complexes averaged 10 Å^2^ in the SASA plot which is also confirmed by the probability distribution plot. The exposure of core residues to the surrounding solvent is minimal which is an additive property to protein stability. The formation of a hydrophobic pocket around both residues also provides confirmation for the lower SASA value (Figure 6(d)).

### 3.5. Secondary Structure Count and Interaction Dynamics

44.39% of amino acids of 3BBT when bound to isocolumbin took part in secondary structure formation, while with isopropylideneandrographolide the percentage reduced to 41.03% which can be targeted toward the increased SASA value.

A difference of 1% can be observed in the 2IOG protein with two different ligands. With 14-acetylandrographolide, a total of 58.08% of residues formed secondary structures, while the number decreased to 57.07% with isopropylideneandrographolide (Table 4).

To assess the binding of protein-ligand complexes, protein-ligand contact estimation and analysis become crucial.

**Figure 7:**
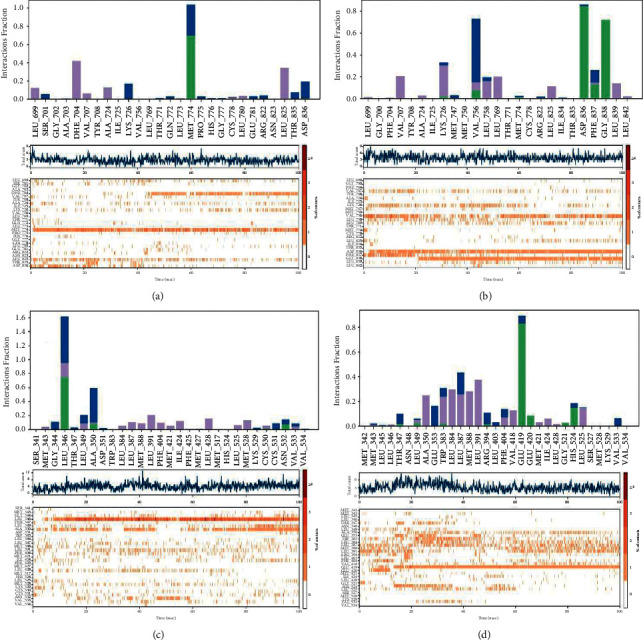
Interaction analysis of (a) 3BBT- isopropylideneandrographolide, (b) 3BBT-isocolumbin, (c) 2IOG-isopropylideneandrographolide, and (d) 2IOG-14-acetylandrographolide.

Considering the 3BBT-isocolumbin complex (Figure 7(b) ), ASP836 and GLY838 anchored the ligand molecules with multiple contacts, and both formed significant string hydrogen bonds. They are assisted by the VAL756 residue, which forms the major water bridges. A total of 25 residues interacted with the ligand at different instances. LYS726, PHE837, LEU758, and VAL756 formed multiple types of bonds. The timeline graph showed a well-complemented scattered binding with only 1 instant of no binding. The other complex with isopropylideneandrographolide (Figure 7(a)) interacts with 27 amino acids, but only only MET774 acts as the anchor to the ligand. The remaining amino acids interacted with the ligand in a scattered and faded manner with a lot more instances of zero contact with the ligand.

Two 2IOG complexes showed quite significant interactions in their own sense. The complex with 14-acetylandrographolide had 31 total residues that interacted with it. GLU419 forms a strong hydrogen bond along with HIS524 and a few more residues. ALA350, TRP383, LEU387, MET388, LEU391, PHE404, VAL418, and LEU525 (Figure 7(c)) stabilized the protein-ligand complex using hydrophobic interactions and contributed to the retention of the ligand in the binding pocket. On the other hand, with isopropylideneandrographolide, LEU346 formed major interactions by showing 3 types of binding, i.e., hydrogen bonds, hydrophobic bonds, and water bridges. The LEU346 interaction is briefly assisted by various residues such as ALA350, LEU383, LEU391, LEU 428, MET525, MET421, PHE404, and MET388 (Figure 7(d)). In the beginning, a lot of instances can be observed having no contact with the ligand which may explain the higher RMSD in the beginning.

In the last decade or so, virtual screening of ligand libraries has proven to be a quite effective methodology for aiding research in the therapy of various ailments. Breast cancer is one such ailment which predominantly affects women. Developing a lead molecule for breast cancer is important and also interesting, considering the complex molecular nature. In this investigation, the computational-based screening defines the initial steps toward the development of a lead compound. Numerous other studies have shown the essentiality of the protein molecules taken in this study, i.e., the estrogen receptor and HER4 protein. Recently, phytochemicals have been an area of interest for all drug formulation experts, and a similar interest drove us to explore the compounds known from five plants which are under investigation for their anticancer effects. *Beet vulgaris* has been of interest to many researchers, and they have successfully shown its cytotoxic activities [38] against various tumors such as liver, skin, and lung tumors [38–41]. Similar activity is shown by *Ocimum sanctum* extract against leukemic cell lines [42], while *Tinospora cordifolia* is being extensively studied. It modulated multiple pathways in colon cancer to prevent its proliferation and growth [43]. Some investigations regard *Andrographis paniculata* as a miracle folk plant for treating cancer. Studies have concluded its extract puts a stopper to cell growth and reduces the chromosomal aberrations [44]. In certain cell lines, the extract also influenced the inflammatory pathways by inactivating NF-kB pathways [45]. Our study, coincidentally, concluded to find two potential compounds extracted from *Andrographis paniculata* alone and the other from *Tinospora cordifolia.* Our study highlights the particular compounds present in the extracts which can specifically target the breast cancer proteins. The results are even suggestive of a multitarget drug molecule, i.e., isopropylideneandrographolide.

## 4. PASS Webserver Prediction

The webserver can be used to predict the biological activity based on the ligand's structure. The three shortlisted compounds in the study were subjected to the prediction. All of the compounds led to the same biological activity. The probability of the ligands acting as antineoplastics, i.e., drugs having tumor restrictive property, ranged between 0.882 and 0.959 when Pa > Pi (Table 5).

## 5. Conclusion

The aim of this study was to identify natural compounds that can prove effective against different proteins associated with breast cancer with few or no side effects. Sixty-two compounds from five selected plants were shortlisted for this study, out of which eighteen compounds were ruled out in violation with Lipinski's rule of five. The least binding affinities and corresponding binding poses for the remaining forty-four compounds were determined. Thereafter, MD simulation studies were carried out on the two best docked complexes for each receptor taken into account, followed by prediction of biological activity of the lead compounds. The results revealed that isocolumbin, isopropylideneandrographolide, and 14-acetylandrographolide are lead compounds against selected breast cancer proteins. All these compounds have antineoplastic effects. Further research should be encouraged to determine the in vitro efficacy of the lead compounds and their exact mechanism of action.

## Figures and Tables

**Table 1:** Molecular docking analysis with 2IOG.

Compounds	Binding energy	Ligand efficiency	Inhibition constant (*μ*M)	Intermolecular energy	Vdw H bond desolvation energy
Isopropylideneandrographolide	−10.3	−0.37	0.02811	−11.2	−11.05
14-Acetylandrographolide	−9.26	−0.33	0.16439	−11.34	−11.37
(8S,12 R)-Isoandrographolide	−9.2	−0.37	0.18169	−10.39	−10.07
Dehydroandrographolide	−9.07	−0.38	0.22425	−10.56	−10.15
Isocolumbin	−9.05	−0.35	0.23209	−9.65	−9.58
Andrographolide	−8.87	−0.35	0.31251	−10.66	−10.38
Palmarin	−8.79	−0.33	0.36334	−9.38	−9.45
19-O-Acetyl-14-deoxy-11,12-didehydroandrographolide	−8.72	−0.32	0.40811	−10.51	−10.46
14-Deoxy-11,12-didehydroandrographolide	−8.48	−0.35	0.60911	−9.97	−9.87
Azatoxin	−8.41	−0.3	0.68172	−9.61	−9.61
Podophyllotoxin	−8.38	−0.28	0.72584	−9.87	−9.77
4-Demethylpodophyllotoxin	−8.35	−0.29	0.76082	−9.84	−9.86
Tinosporin	−8.28	−0.29	0.85158	−9.77	−9.79
Palmatine	−8.26	−0.32	0.88102	−9.45	−9.44
Tetrahydropalmatine	−8.26	−0.32	0.87684	−9.46	−9.25
Luteolin	−8.1	−0.39	1.16	−9.59	−9.04
Tembetarine	−8.05	−0.32	1.26	−9.84	−9.61
Cirsilineol	−7.89	−0.32	1.42	−9.77	−9.71
Cirsimaritin	−7.81	−0.34	1.9	−9.3	−9.04
Apigenin	−7.76	−0.39	2.06	−8.95	−8.57
Jatrorrhizine	−7.75	−0.31	2.08	−8.95	−9
Rhamnetin	−7.64	−0.33	2.5	−9.43	−8.93
Magnoflorine	−7.62	−0.3	2.59	−8.81	−8.43
Aporphine	−7.61	−0.42	2.63	−7.61	−7.25
Quercetin	−7.61	−0.35	2.65	−9.4	−8.87
Isothymusin	−7.41	−0.31	3.67	−9.2	−8.95
Kaempferol	−7.39	−0.35	3.85	−8.88	−8.53
Moslosooflavone	−7.17	−0.33	5.57	−8.36	−8.3
Rosmarinic acid	−7.16	−0.28	5.63	−10.74	−10.42
Rhamnocitrin	−7.12	−0.32	6.06	−8.61	−8.34
*alpha*-Elemene	−6.89	−0.46	8.9	−7.49	−7.49
Betaxanthin	−6.88	−0.26	9.06	−9.27	−9.61
Myrtenal	−6.44	−0.59	19.15	−6.73	−6.46
Bornyl acetate	−6.25	−0.45	26.32	−6.84	−6.82
Eugenol	−5.49	−0.46	94.23	−6.69	−6.61
Methyl eugenol	−5.26	−0.4	138.4	−6.46	−6.27
Berberin	−5.25	−0.21	141.76	−5.85	−5.83
Ferulic acid	−5.24	−0.37	144.6	−6.73	−5.47
Neral	−5.19	−0.47	157.53	−6.38	−6.24
p-Coumaric acid	−5.09	−0.42	186.95	−6.28	−4.94
Caffeic acid	−5.08	−0.39	190.47	−6.57	−6.19
Syringic acid	−4.86	−0.35	274.22	−6.35	−5.78
Betaine	−3.6	−0.45	2310	−4.19	−2.83
Choline	−3.16	−0.45	4810	−4.06	−3.63

**Table 2:** Molecular docking analysis with 3BBT.

Compounds	Binding energy	Ligand efficiency	Inhibition constant (*μ*M)	Intermolecular energy	Vdw H bond desolvation energy
Isopropylideneandrographolide	−9.41	−0.34	0.12718	−10.3	−10.2
Isocolumbin	−9.06	−0.35	0.22848	−9.66	−9.46
Azatoxin	−8.9	−0.32	0.30049	−10.09	−9.87
19-O-Acetyl-14-deoxy-11,12-didehydroandrographolide	−8.84	−0.33	0.329	−10.63	−10.48
Kaempferol	−8.18	−0.39	1	−9.68	−9.41
(8S,12 R)-Isoandrographolide	−8.11	−0.32	1.13	−9.31	−9.27
14-Acetylandrographolide	−8.1	−0.29	1.16	−10.19	−10.04
14-Deoxy-11,12-didehydroandrographolide	−7.97	−0.33	1.43	−9.46	−9.31
Cirsilineol	−7.95	−0.32	1.49	−9.74	−9.38
Apigenin	−7.95	−0.4	1.49	−9.14	−9.06
Betaxanthin	−7.93	−0.31	1.53	−10.32	−8.98
Rhamnetin	−7.88	−0.34	1.68	−9.67	−9.57
Andrographolide	−7.87	−0.31	1.69	−9.66	−9.39
4-Demethylpodophyllotoxin	−7.76	−0.27	2.06	−9.25	−8.96
Luteolin	−7.68	−0.37	2.35	−9.17	−8.82
Berberin	−7.6	−0.3	2.69	−8.2	−8.13
Palmatine	−7.6	−0.29	2.67	−8.8	−8.7
*alpha*-Elemene	−7.57	−0.5	2.82	−8.17	−8.18
Dehydroandrographolide	−7.56	−0.32	2.86	−9.06	−8.7
Rhamnocitrin	−7.54	−0.34	2.95	−9.04	−8.99
Palmarin	−7.5	−0.28	3.19	−8.09	−8
Quercetin	−7.48	−0.34	3.3	−9.27	−9.18
Moslosooflavone	−7.47	−0.34	3.33	−8.67	−8.93
Cirsimaritin	−7.33	−0.32	4.21	−8.83	−8.53
Podophyllotoxin	−7.32	−0.24	4.31	−8.81	−8.83
Rosmarinic acid	−7.2	−0.28	5.26	−10.78	−10.09
Magnoflorine	−7.09	−0.28	6.4	−8.28	−8.32
Tetrahydropalmatine	−6.99	−0.27	7.47	−8.19	−8.48
Jatrorrhizine	−6.96	−0.28	7.86	−8.16	−8.2
Isothymusin	−6.91	−0.29	8.64	−8.7	−8.42
Bornyl acetate	−6.88	−0.49	9.09	−7.47	−7.36
Myrtenal	−6.52	−0.59	16.71	−6.82	−6.84
Tembetarine	−6.48	−0.26	17.84	−8.27	−8.35
Tinosporin	−6.19	−0.21	28.94	−7.68	−7.65
Caffeic acid	−6.11	−0.47	33.16	−7.6	−6.32
Ferulic acid	−5.85	−0.42	51.48	−7.34	−6.23
Neral	−5.8	−0.53	56.3	−6.99	−6.88
p-Coumaric acid	−5.79	−0.48	57.24	−6.98	−5.72
Methyl eugenol	−5.73	−0.44	63.14	−6.92	−6.79
Syringic acid	−5.41	−0.39	107.55	−6.91	−6.22
Eugenol	−5.28	−0.44	134.03	−6.48	−6.46
Aporphine	−5	−0.28	216.99	−5	−5.44
Betaine	−3.69	−0.46	1980	−4.28	−2.67
Choline	−2.91	−0.42	7360	−3.81	−3.93

**Table 3:** Potential and total energies (kcal/mol) of systems obtained after 100 ns simulation.

S. no.	Complex	Potential energy (kcal/mol)	Total energy (kcal/mol)
1.	3BBT-Isopropylideneandrographolide	−166930.943	−205596.718
2.	3BBT-Isocolumbin	−166941.883	−205610.056
3.	2IOG-Isopropylideneandrographolide	−85478.448	−105151.438
4.	2IOG-14-Acetylandrographolide	−85481.496	−105151.587

**Table 4:** Protein secondary structure estimation.

Complex	Helix (%)	Strand (%)	Total (%)
3BBT-Isopropylideneandrographolide	27.54	13.49	41.03
3BBT-Isolcolumbin	31.00	13.39	44.39
2IOG-14-Acetylandrographolide	55.23	2.86	58.08
2IOG-Isopropylideneandrographolide	54.08	2.99	57.07

**Table 5:** Biological activity prediction of the selected three ligands.

Compound	Pa	Pi	Biological activity prediction
Isocolumbin	0.882	0.005	Antineoplastic
Isopropylideneandrographolide	0.954	0.004	Antineoplastic
14-Acetylandrographolide	0.959	0.004	Antineoplastic

Pa = probability to be active; Pi = probability to be inactive.

## Data Availability

The data used to support the findings of this study are included within the article.
